# Supplementary material for: Analysing interventions designed to reduce tuberculosis-related stigma: A scoping review
Source: PLOS Glob Public Health. 2022 Oct 19;2(10):e0000989. doi: 10.1371/journal.pgph.0000989 (PMC10022226; doi:10.1371/journal.pgph.0000989)
Supplement: S1 Appendix — (DOCX) [file pgph.0000989.s001.docx]

**APPENDIX**

**TB stigma search terms:**

("Tuberculosis/psychology*"[MeSH] OR "Tuberculosis"[tiab] OR "tubercul*"[tiab] OR "TB"[tiab] OR "pulmonary consumption" [tiab] OR "Tuberculosis, Pulmonary"[MeSH] OR "multidrug-resistant tuberculosis" [tiab] OR "Tuberculosis, Multidrug-Resistant"[MeSH]) AND ("stigma*"[tiab] OR "TB stigma"[tiab] OR "social stigma*"[MeSH] OR "Social Stigma"[tiab] OR ("social"[tiab] AND "stigma"[tiab]) OR "self stigma*"[tiab])

**TB stigma intervention search terms:**

("Tuberculosis/psychology*"[MeSH] OR "Tuberculosis"[tiab] OR "tubercul*"[tiab] OR "TB"[tiab] OR "pulmonary consumption" [tiab] OR "Tuberculosis, Pulmonary"[MeSH] OR "multidrug-resistant tuberculosis" [tiab] OR "Tuberculosis, Multidrug-Resistant"[MeSH]) AND ("stigma reduction interventions"[tiab] OR "Stigma reduction"[tiab] OR "Social disapproval"[tiab] OR "Social Withdrawal"[tiab])
