# Supplementary material for: Analysing interventions designed to reduce tuberculosis-related stigma: A scoping review
Source: PLOS Glob Public Health. 2022 Oct 19;2(10):e0000989. doi: 10.1371/journal.pgph.0000989 (PMC10022226; doi:10.1371/journal.pgph.0000989)
Supplement: S3 Appendix — (DOCX) [file pgph.0000989.s003.docx]

Assessment of implementation strategy and outcomes for included studies (n=9)

| Implementation details reported | Total (%) |
| --- | --- |
| Actor | 7 (77.8%) |
| Action | 9 (100%) |
| Target of the action | 9 (100%) |
| Temporality | 6 (66.7%) |
| Dose | 7 (77.8%) |
| Justification | 3 (33.3%) |
| Implementation outcomes reported | **Total (%)** |
| Acceptability | 4 (44.4%) |
| Adoption | 3 (33.3%) |
| Appropriateness | 7 (77.8%) |
| Cost | 3 (33.3%) |
| Feasibility | 5 (55.6%) |
| Fidelity | 4 (44.4%) |
| Penetration | 1 (11.1%) |
| Sustainability | 3 (33.3%) |
