# Supplementary material for: Analysing interventions designed to reduce tuberculosis-related stigma: A scoping review
Source: PLOS Glob Public Health. 2022 Oct 19;2(10):e0000989. doi: 10.1371/journal.pgph.0000989 (PMC10022226; doi:10.1371/journal.pgph.0000989)
Supplement: Attachment — Submitted filename: Stigma scoping review_Response to reviewers_4.4.22.docx [file pgph.0000989.s005.docx]

**Analysing interventions designed to reduce tuberculosis-related stigma: a scoping review.**

**Response to reviewer comments**

Reviewers' comments:

Reviewer's Responses to Questions

**Comments to the Author**

1. Does this manuscript meet PLOS Global Public Health’s [publication criteria](https://urldefense.com/v3/__https:/journals.plos.org/globalpublichealth/s/criteria-for-publication__;!!CvMGjuU!vYGzTMwoaat5VRyvSaPuRBwXpIQij9A3RPy47_oXkQj9zMmRBhypbTIM8oNbkbqQamlu-VI$)? Is the manuscript technically sound, and do the data support the conclusions? The manuscript must describe methodologically and ethically rigorous research with conclusions that are appropriately drawn based on the data presented.

Reviewer #1: Yes

Reviewer #2: Yes

2. Has the statistical analysis been performed appropriately and rigorously?

Reviewer #1: N/A

Reviewer #2: N/A

3. Have the authors made all data underlying the findings in their manuscript fully available (please refer to the Data Availability Statement at the start of the manuscript PDF file)?

The [PLOS Data policy](https://urldefense.com/v3/__https:/journals.plos.org/globalpublichealth/s/data-availability__;!!CvMGjuU!vYGzTMwoaat5VRyvSaPuRBwXpIQij9A3RPy47_oXkQj9zMmRBhypbTIM8oNbkbqQNqQkMBc$) requires authors to make all data underlying the findings described in their manuscript fully available without restriction, with rare exception. The data should be provided as part of the manuscript or its supporting information, or deposited to a public repository. For example, in addition to summary statistics, the data points behind means, medians and variance measures should be available. If there are restrictions on publicly sharing data—e.g. participant privacy or use of data from a third party—those must be specified.

Reviewer #1: Yes

Reviewer #2: Yes

4. Is the manuscript presented in an intelligible fashion and written in standard English?

PLOS Global Public Health does not copyedit accepted manuscripts, so the language in submitted articles must be clear, correct, and unambiguous. Any typographical or grammatical errors should be corrected at revision, so please note any specific errors here.

Reviewer #1: Yes

Reviewer #2: Yes

5. Review Comments to the Author

Please use the space provided to explain your answers to the questions above. You may also include additional comments for the author, including concerns about dual publication, research ethics, or publication ethics. (Please upload your review as an attachment if it exceeds 20,000 characters)

**We thank both reviewers for their positive feedback and valuable suggestions that we have incorporated in our revised manuscript. Our responses to the reviewers are in bold text below. Please note that all line reference numbers below refer to the R1 manuscript clean version. Quotes from the revised manuscript are written in italics.**

Reviewer #1: This is a very well written review. Findings of this review is in sync with the systematic review by Sommerland et al on the same topic. Authors have highlighted the significance of having a standard definition and measurement of stigma for better implementation.

**We thank the reviewer for this positive feedback.**

Since the authors used their definitions to assess and categorize the stigma measurement indicator across studies, there is the possibility of misclassification. It would be good to explain how this was taken care of.

**1.1 In the Methods, we have now added text to clarify how we approached the definitions of stigma manifestations using recent guidance developed by KNCV and USAID on stigma measurement and provided an example where we reclassified the categorization of a stigma manifestation in an included study based on this. Please see lines 159-165:**

***‘We used recently developed guidance on TB stigma measurement designed with expert consensus input to standardize the definitions used by the reviewers to classify stigma manifestations, rather than definitions used in individual studies to enable comparison across studies. For example, in one study the phrase ‘I stay away from people… to avoid being rejected’ had been classified as internal stigma (21). However, as per the stigma manifestation classification used in this review (38) this indicator was reclassified as a measure of anticipated stigma.‘***

Further it would be prudent if there was a mention of the possible limitations that the study might have had.

**1.2 We have added text on study limitations, please see lines 502-509 and the following response 1.3.**

Since the search was restricted to peer reviewed articles there is the possibility of having missed relevant literature from certain low and middle income countries with significant TB burden. Perhaps this should be looked into

**1.3 We acknowledge that restricting the search to peer reviewed articles may exclude relevant literature as the reviewer points out and have added this as a limitation. We also clarified that the stakeholder consultation facilitated identification of relevant policy documents and cite these. Please see lines 478-479.**

***‘While national documents detailing strategies to end TB stigma exist in countries such as India, stigma remains all too pervasive in practice settings.’***

Reviewer #2: A very good narrative synthesis and relevant to current discourses around stigma mitigation, and will add value to the current discourse on stigma and TB. But more explicit application of the stated ecological framework is needed in categorizing the review findings, and results sections needs more organization to enhance clarity and coherence. Some suggestions:

**We appreciate this positive feedback and have incorporated this valuable suggestion regarding better organization of the results to be aligned with a socio-ecological framework to categorize and evaluate stigma reduction interventions, please see more detailed responses below.**

1. Suggest more explicitly stating how this review differs from / builds on prior reviews on the topic of TB stigma (eg, Sommerland, especially as it is the most recent, but also others such as Courtwright & Turner, Macq, Nyblade, etc). I do see Sommerland mentioned (line 391) but given cursory attention. That review went into substantial detail into measurement methods as well. Suggest highlighting any new studies found through the current search and specifying the ‘additional insights’ thereby attained, as suggested at the very end of the paper (lines 462 464).

**2.1 We have added additional text to describe the primary findings of the key reviews mentioned above (Macq, Courtwright, Nyblade and Sommerland) and how our findings align with these and provide additional insights in lines 400-410.**

***Prior reviews on TB stigma have highlighted the scarcity of published TB stigma assessments and particular need to address internal stigma experienced by people with TB (22), the need to ensure diversity in patient populations assessed for stigma and use of validated survey tools to quantify the impact at different steps in the TB cascade of care (36), and the lack of interventions to address TB related stigma in health facilities (37). We note that four further studies have been included in this review, compared to the most recent prior review (8), and that these studies include stigma interventions targeted towards health workers rather than focusing only on people with TB. While our findings align with prior reviews regarding the low number and quality of published studies on this topic, we provide additional insights based on applying a socio-ecological framework to categorize interventions, evaluating implementation processes and outcomes, and conducting a stakeholder consultation to contextualise our findings.***

2. At least one study was an RCT, and worthy of mention in the quality section.

**2.2 We re-reviewed our quality assessment data and have now accordingly upgraded the Bond RCT and revised the text, please see lines 201-202:**

***‘One study that was a randomized controlled trial (although stigma was not a primary outcome) scored 100%.’***

3. The categories of interventions are either not defined or then not ideally utilized. For example, no definitions for “support groups”, information” and “counselling” are provided; these would be crucial and should not be assumed. Also, while the classifications of individual, interpersonal and community are provided, the subsequent allocation of interventions into each does not seem accurate. Support groups are classified as individual level interventions but they have a strong interpersonal element. Indeed, the researchers’ own definition (line 276) applies to support groups (“reinforcing social networks” – a peer network is a valued social network). See next point as well.

**2.3 We appreciate this point and the following one that is related. We have reclassified the interventions according to the socio-ecological level at which the intervention is targeted (individual, interpersonal, organizational, community, and policy) and provided detailed definitions for these levels in the Methods section within lines 147-159. Given that we changed the intervention categorization, we did not aim to provide standardized definitions for support groups, information and counselling, since these are heterogeneously implemented in practice, but rather explained how these were delivered in a given intervention. We agree that some interventions targeted more than one socio-ecological level and have categorized this accordingly in Table 1 and the corresponding text, please see our revised Results section.**

4. Having these two axes or classifications for interventions (ie, individual, interpersonal, community and support groups, information based, counselling) is weakening coherence of this otherwise very important paper. The authors would be recommended to consolidate (ie, pick one framework for classification, and subsume the other into it). As the ecological model is already explicitly stated as a desired framework, the former classification of individual-interpersonal-community would work. What is missing is structural or policy level, and should be remarked within the discussion if no papers in this regard were located.

a. Accordingly, classification/categorization of interventions in Table 1 should change to be individual, interpersonal and community.

**2.4 We have now recategorized our results according to a single framework – the Health Stigma and Discrimination framework, and have accordingly restructured Table 1 and the results to discuss the studies according to the socio-ecological level at which the intervention is targeted. We note the lack of identified policy level interventions in the discussion lines 396-398.**

***‘No interventions targeted structural or policy level changes, although the adaptation of policies and practices that perpetuate TB stigma has been identified as a core tenet for the elimination of TB stigma (35).’***

5. Information in Tables 1 and 2 should be reconciled and consolidated. For example: Table 1 should have columns on measurement tools and approaches. The final column of Table 1 at this time has no information on the construct/measure (eg, anticipated stigma, driver of stigma) that was addressed/changed. By combining measurement information from Table 1 and 2, this crucial detail will automatically become more clear to a reader.

**2.5 We have now combined Tables 1 and 2, such that the measurement tools and approaches have been incorporated into Table 1 for clarity.**

6. Table 3. It is unclear what value it adds to the discussion given that the studies in which those measures were assessed is not shared. The information, if imperative, may be added to Table 1. Otherwise, the table can be moved to a supplementary file.

**2.6 We have revised the text on intervention implementation approach and evaluation which we think provides important context for readers including future intervention developers. As suggested, we have moved Table 3 into our supplementary file.**

7. Under “Intervening at the Individual level”, what is the subtitle “stigma manifestations” alluding to? The same goes for the from the authors’ review. As an example, rather than listing other sub-titles (stigma drivers and manifestations) within subsequent two sections. An introductory statement is needed to tie in the text with each of these subtitles, and thereafter a more authentic narrative synthesis is needed to ascertain what exactly the reader should take studies from Peru, Nicaragua and South Africa/Zambia one after the next (Line 241 onward), perhaps use your analytic framework to consolidate the main/overlapping points: Two studies successfully reduced internal stigma. One, from XYZ, utilized [said intervention components]… whereas the other, from ABC, implemented […]. A third study also acting at the individual level, reduced anticipated stigma…

**2.7 We have now extensively revised our Results section based on these and earlier suggestions such that we primarily classify interventions based on their socio-ecological level and secondarily examine their impact on specific stigma manifestations or drivers as per the Health Stigma and Discrimination framework. We think that the latter subsections indicating which stigma manifestation (internal, anticipated, enacted) or stigma drivers (e.g. blame, misconceptions) was targeted by a given intervention will be helpful to readers to understand how different interventions can impact different manifestations and drivers of TB stigma, which is important since effective interventions need to be context specific. Nonetheless, we have provided introductory statements for each of these subsections and consolidated the individual study findings to create authentic narrative synthesis as suggested, please see the revised Results section, lines 242-384.**

8. The terms domains and manifestations are used to describe the same concept (Fig 2 and results uses manifestations, whereas methods and discussion uses domains). Suggest more consistency.

**2.8 Since we are using Stangl et al.’s Health Stigma and Discrimination framework as the overarching framework to categorize and evaluate the interventions we have identified, we have used the term stigma manifestation, as per this framework and ensured consistency when we discuss internal, anticipated, and enacted stigma, and stigma drivers when we discuss aspects such as blame which is a recognized driver for the manifestation of internal stigma.**

9. Line 340 – may be clearer to state the intervention was successful in only one setting.

**2.9 We have now revised this text to clarify that the effect of the stigma intervention in Bond et al was only successful in one setting, please see lines 365-368.**

***‘TB related stigma was found to be highly contextual, as demonstrated when the same intervention applied to two study sites in different countries and was successful in one but not the other.’***

10. In this same section, it would be helpful to have a quick summary of what the main barriers were (even if they were only reported in 2-3 studies). There is a final implication suggested for just one of the barriers (mental health) but not the other barrier (discriminatory attitudes of staff).

**2.10 We have now summarized the main barriers to implementation in lines 365-380 and added the implications of these barriers in lines 369-371 and 381-384.**

***‘Recruiting and appropriately training intervention facilitators and addressing mental health in people with TB were identified as important barrier across several studies.’***

***‘Analysis of these barriers highlights the need to ensure high-quality training for staff delivering interventions in studies and moreover to programmatic staff delivering TB care, and for integrated mental health services to be available for people with TB, in addition to interventions targeting stigma.’***

11. The stakeholder consultation is commendable but the process and purpose remains unclear. Why was the consultation held in South Africa alone (what are the limits thereof), and how many individuals were engaged? It is unclear if stakeholders’ inputs informed the authors’ review/ narrative synthesis; suggest being explicit about this. If they were engaged, more language about their participation in analysis should be shared. If they were not engaged in analysis but rather served as a final space for exchanging feedback, then the entire consultation and ideas emanating thereof may be better situated within the discussion alone (eg, as a knowledge exchange strategy). To name stakeholders as participants suggests a new research study was undertaken, so suggest using more apt terms to describe the stakeholders involved.

**2.11 We have clarified that this review was conducted as part of a community-engaged research study to evaluate TB stigma using community-based stigma assessments in South Africa and use these findings to develop stigma reduction interventions, please see lines 469-481, which explains why this consultation was held with South African stakeholders alone. We have also added text about the limitations of conducting the stakeholder consultation only in South Africa in lines 504-506.**

**The stakeholder consultation was held as an exercise to exchange feedback. Stakeholders were not engaged in analysis of the results, but rather to supplement results and recommend emergent priorities for future research by drawing on their expertise and experiences. This is a recommended stage of scoping reviews (Arskey & O’Malley., 2005). To ensure this is clear, as recommended by the reviewer, reporting of the stakeholder consultation has been moved to the discussion section (lines 469-481) and the methodology has been edited to reflect this (lines 139-144). Stakeholders are no longer referred to as participants in any context in the review.**

12. Given this consultation, did the authors explore the consultation or engagement of community actors within the 9 studies reviewed? Is there space to comment on their inclusion (or exclusion) in the process of intervention design, implementation and/or evaluation?

**2.12 Using the socio-ecological levels for classification, we have added a section on community-level interventions, please see lines 341-351. We have added reference to the studies which included consultation with community actors in the ‘Implementation Details Evaluated’ section (specifically in lines 238-240).**

***‘Although only one intervention was at the community-level and used community volunteers as part of the implementation team (23), several others used community actors such as household counselors (25), or CHWs to deliver the intervention and local leaders to provide support (27).’***

**We have also added text in the discussion related to the stakeholder consultation in lines 487-491.**

***‘Additional actors both within the healthcare system and in the community should also be engaged to reduce stigma, who may include traditional healers and church leaders who often overlook key entry points to the TB cascade of care. Interventions that equip these groups with tools to mitigate stigma drivers may have great potential to reduce stigma manifestations.’***

13. Several of the studies were undertaken in settings with high TB-HIV co-prevalence where there was likely overlapping stigma. Yet there is no remark about this in the review analysis. It is mentioned by a stakeholder. The authors need to connect the stakeholder consultation to the review findings.

**2.13 We agree that intersectional stigma is an important barrier to quality TB care in high TB-HIV incidence countries and should be addressed in intervention design, as suggested by our stakeholder consultation. We have accordingly added text to the Discussion section in lines 419-420:**

***‘Further, in communities with high co-prevalence of HIV and TB, intersectional stigma is common, as noted in several included studies (25, 29, 30), and should be addressed in intervention design.’***

14. Points stated in lines 407-409 are crucial. Is there any reference or connection to such findings from other disease states or research projects that could develop this point further? It would be a missed opportunity is authors do not raise the issue of power, inequity and/or the hegemonic dimensions of stigma, even if briefly.

**2.14 We thank the reviewer for this important point that expands upon our previous statement about addressing conscious and unconscious biases through high-quality training for those delivering interventions to avoid perpetuating and spreading stigma. We have added text to develop these points further as suggested by the reviewer, please see lines 424-31.**

***‘Important implementation barriers to overcome include the need for high-quality training regarding stigma and/or TB for those implementing intervention that should address conscious and unconscious biases, since negative attitudes toward people with TB can otherwise be perpetuated and spread by those delivering the intervention.***

***Moreover, intervention designers and implementers should reflect on and aim to address the broader hegemonic dimensions of stigma, which often reflect societal inequities and racism that mediate the risk of acquiring TB and poor TB outcomes, and also serve as drivers for exacerbated and intersectional stigma.’***

6. PLOS authors have the option to publish the peer review history of their article ([what does this mean?](https://urldefense.com/v3/__https:/journals.plos.org/globalpublichealth/s/editorial-and-peer-review-process*loc-peer-review-history__;Iw!!CvMGjuU!vYGzTMwoaat5VRyvSaPuRBwXpIQij9A3RPy47_oXkQj9zMmRBhypbTIM8oNbkbqQSqwr18A$)). If published, this will include your full peer review and any attached files.


**Do you want your identity to be public for this peer review?** If you choose “no”, your identity will remain anonymous but your review may still be made public.


For information about this choice, including consent withdrawal, please see our [Privacy Policy](https://urldefense.com/v3/__https:/www.plos.org/privacy-policy__;!!CvMGjuU!vYGzTMwoaat5VRyvSaPuRBwXpIQij9A3RPy47_oXkQj9zMmRBhypbTIM8oNbkbqQ9HosNzE$).

Reviewer #1: No

Reviewer #2: No

[NOTE: If reviewer comments were submitted as an attachment file, they will be attached to this email and accessible via the submission site. Please log into your account, locate the manuscript record, and check for the action link "View Attachments". If this link does not appear, there are no attachment files.]

While revising your submission, please upload your figure files to the Preflight Analysis and Conversion Engine (PACE) digital diagnostic tool, [https://pacev2.apexcovantage.com/](https://urldefense.com/v3/__https:/pacev2.apexcovantage.com/__;!!CvMGjuU!vYGzTMwoaat5VRyvSaPuRBwXpIQij9A3RPy47_oXkQj9zMmRBhypbTIM8oNbkbqQYMK05Hs$). PACE helps ensure that figures meet PLOS requirements. To use PACE, you must first register as a user. Registration is free. Then, login and navigate to the UPLOAD tab, where you will find detailed instructions on how to use the tool. If you encounter any issues or have any questions when using PACE, please email PLOS at figures@plos.org. Please note that Supporting Information files do not need this step.

Journal Requirements:

1. Boxes should be editable and included within the manuscript file like tables. Please amend this.

**We have done this.**

2. We have noticed that you have uploaded supporting information but you have not included a list of legends.  Please add a full list of legends for all supporting information files (including figures, table and data files) after the references list.

**We have added this.**

3. In the online submission form, you indicated that "All individuals studies analyzed in this scoping review are publicly available. The aggregate datasets used and analysed during the current study are available from the corresponding author on reasonable request.".

**We have provided our data extraction form as a supplementary file.**

All PLOS journals now require all data underlying the findings described in their manuscript to be freely available to other researchers, either 1. In a public repository, 2. Within the manuscript itself, or 3. Uploaded as supplementary information.
This policy applies to all data except where public deposition would breach compliance with the protocol approved by your research ethics board. If your data cannot be made publicly available for ethical or legal reasons (e.g., public availability would compromise patient privacy), please explain your reasons by return email and your exemption request will be escalated to the editor for approval. Your exemption request will be handled independently and will not hold up the peer review process, but will need to be resolved should your manuscript be accepted for publication. One of the Editorial team will then be in touch if there are any issues.

4. Please amend your detailed Financial Disclosure statement. This is published with the article, therefore should be completed in full sentences and contain the exact wording you wish to be published.

iii). State what role the funders took in the study. If the funders had no role in your study, please state: “The funders had no role in study design, data collection and analysis, decision to publish, or preparation of the manuscript.”

**We have added this.**

Additional Editor Comments (if provided):
